# Repetition of Dose*Read before the Central N. Y. Homœopathic Medical Society, at Syracure, December 15th, 1880.

**Published:** 1882-03

**Authors:** E. B. Nash

**Affiliations:** Cortland, N. Y.


### THE REPETITION OF THE DOSE.*

E. B. Nash, M. D. Cortland, N. Y.

One would naturally suppose that but little that is new can now be said on this subject. The three cardinal principles, viz:—the similars; the single remedy, and the minimum dose, are generally accepted by homœopathists; and this subject of the repetition of the dose is embraced in the latter. If it be true that the minimum in one case is not the minimum in another, or in other words, it is necessary to use the tincture in one case (as did Hahnemann), and the CM in another; then why is it not likely to be true, that undue or too frequent repetition would be as much out of place as prescribing the 3rd when the 200th was the minimum, and *vice versa?*

It is well understood that the primary effect of a drug prescribed homœopathically, acting in the direction of the disease as it does, must be to cause (even though it be so slight as to be imperceptible) a temporary aggravation. Hahnemann teaches that the dose must be large enough to produce this aggravation in order to be curative (§279 and 280).

---

* Read before the Central N. Y. Homœopathic Medical Society, at Syracure, December 15th, 1880.

Again, it is well understood, that it is the secondary effect or reaction that *cures.* This being true, the violence and duration of the primary effect, of course, must pass away before the cure begins. What, then, would be the result if the dose were too large, or too frequently repeated? Evidently, in the first case, unnecessary suffering from too intense aggravation; in the second, hindered reaction or *cure.* These truths seem self-evident.

Then it seems to me that there can be but one correct rule, and that is, *to repeat the dose when reaction* (curative action) *ceases.* How frequently the dose must be repeated on account of expended reaction, must depend upon both the individual and the disease with which we have to do.

As some individuals are more susceptible to drug action than others, and require the small or smallest dose to affect them, so some are possessed of stronger reactive power, and the response to the proper remedy will be more lasting. Violent, acute diseases, attacking persons in full vigor and progressing rapidly, call for more frequent repetition of the remedy than chronic diseases. Hahnemann speaks in the paragraph above referred to of the proper dose being able to cure a *portion* of the disease. Every dose just sufficient is followed by reaction if it is administered after reaction ceases, or where *disease action* is in the ascendency. If administered in sufficient doses to *impress* either in health or during reaction, its effect is *primary*, in the first condition (health) causing disease action, in the latter (reaction) hindering the cure.

It may take more than one dose, especially if the first dose is not large enough, to get the impression that always resides in the homœopathic remedy; but just as soon as that impression is obtained, the remedy should be discontinued so long as improvement continues.

A remedy may, and often has been repeated, after sufficient has been administered to set up curative action, and the result is, the case is either made worse, held *in statu quo*, or recovers more slowly than it would have done if the remedy were properly given. The habit of *doing something*, so long as the patient is not quite well, even though he may be convalescent, is often productive of harm.

*Sac. Lac.* is the remedy when reaction is fully established, and every physician should have plenty of it and *use it.*

It requires as much skill to know when *not* to give medicine, as when to give.

In very acute diseases it may be necessary to repeat very often until an effect is evident. In chronic diseases, improvement once begun, may continue from a single dose even for months, or until cure. We should always remember that *reaction* cures our patient; not the primary action; primary action is always in the direction of the disease; while *reaction* only is in the direction of health.

The allopath gives large doses of *Mandrake* or *Rhubarb* or *Jalap* in constipation for its primary action, and reaction is always defeating him, and reaction in his case is still in the direction of disease as before. And this reaction of which we talk, is nature's effort in the direction of *health*, whether opposed to drug or natural disease, if there is such a thing as natural disease.

Drugs are poisons, and just as sure as you introduce one into the organism, unless the dose is so large as to completely overpower nature, she immediately brings all her forces into action to repel the intruder, and she will continue to strive until entirely overpowered by the drug or until health is established.

Now, a much smaller dose will excite this reaction in disease, when administered homœopathically, than in health, simply because the *disease* renders the part acted upon more susceptible to the drug.

In regard to the *modus operandi* of the homœopathic remedy in curing disease, I can do no better than refer you to § 282 of the *Organon* (Wesselhœft's translation).

You remember the case of colicodynia reported by Hahnemann. It was cured by *Verat. alb.* in 4 gr. doses. The aggravation was so intense that, to use the patient's own expression, he "wrestled with death," *covered with cold sweat*, and almost suffocated. The remedy was homœopathic and a cure resulted; but it might just as surely and more promptly have followed the minimum dose, and saved the terrible suffering. Hahnemann learned a lesson from the case, and was *forced*, as we are, to accept the fact that, to perform the safest, quickest, and pleasantest cure, we must seek to administer a dose just sufficient, "simply this and nothing more;" and repetition has as much to do with it as the *size* of the single dose or the potency.

